# A letter to the Master Clinician

**DOI:** 10.12688/f1000research.3-1.v2

**Published:** 2014-02-13

**Authors:** Kenar D. Jhaveri

**Affiliations:** 1 Department of Internal Medicine, Division of Kidney Diseases and Hypertension, Hofstra North Shore LIJ School of Medicine, Great Neck, NY 11021, USA

## Abstract

In this commentary, the author writes a letter to the Master Clinician about his concerns regarding the teaching responsibilities of current faculty members during ward rounds. This short essay highlights the transition that has been noticed in medical training in the last decade.

Dear *Master Clinician*,

Re: Invitation

I am writing this letter to ask you to please return to us. In your absence, we have felt your loss.

Today, when I walk down the halls of my academic hospital, I feel an emptiness. The constant flow of work onto the secretary’s desk begins at 7 am. The transporter brings a patient back from the ultrasound room. The residents hurry to gather data from the chart before their attending arrives. The nurses scurry to change shifts. These long halls are busy with secretaries working hard, nurses doing their jobs, and nurse practitioners and physician assistants writing notes on the chart. In the midst of all this busy life for our residents, fellows and medical students, there is something now missing.

As a consultant on the floor, I see a crowd of physicians making their rounds. The medical students are easily recognizable by their short white coats. Then I stop, I see someone presenting data: Ahhh! that must be the intern. There are residents discussing patients with a hospitalist. The endocrine consultant team walks onto the floor, and the cycle starts all over again. I have noticed in the last few years that the physician-in-charge is usually inexperienced, and probably has just 1–3 years more experience than the third year resident. What has changed? What is missing? Few now stay on to continue to build their experience but rather join a fellowship program or outpatient practice. Soon, they are replaced by new fresh group of them just graduating from residency. Have we lost the Master Clinician?

The people who inspired us to become who we are today were the great Master Clinicians of their time and they shared with us their wisdom, knowledge and wealth of experience. It would be wrong to say that we are *losing* the art of physical exam and diagnosis, but rather that we have *lost* the art. *You* shared your wisdom with the team and taught the fellows and residents not only bedside manners, but also told us about your experiences. Now *you* have been replaced with inexperienced faculty, textbooks have been replaced by *Google* and stethoscopes have been replaced by handheld devices.

Where have you ** been? Now you only occasionally sit with fellows and residents and give a lecture and share your wisdom. It is always an honor and pleasure to meet someone of your caliber but one might never see you on the hospital floor showing your magic. Why is that? You have taken on extensive administrative roles, spend more time in the laboratory and have less time to come and join us on the wards. While we understand your needs and desires to do other tasks, I wish that you would come back and share your wealth with us on the floor more often. Once a year, I see you come and do some time on the wards; I get very excited that you are able to give us that time. But that time is fragmented by meetings. You have cut short what you do best: teaching, caring for patients and inspiring young professionals. When we lose you to administrative duties, we lose the enthusiasm that you share with young and up-and-coming physicians that creates their passion for medicine. We lose the art of medicine. We lose doctoring…

Why is this Master important in the making of a good clinician? The role of such a person is enormous. A 67 year old male with prostate cancer is admitted for severe metabolic alkalosis, hypokalemia and new onset hypertension. A medical student can spend hours taking histories and performing physical exams, memorize a long list of differential diagnoses but yet not come up with the right diagnosis. This Master Clinician arrives at the correct diagnosis in a few minutes of meeting the same patient. “This is Cushing’s disease-ACTH production from the prostate cancer; start ketaconazole now!” Medical school teaches us the *science of medicine* and post graduate training showcases us the *art of medicine*. From being a good teacher and a great clinician, Master Clinicians such as yourself will demonstrate the *art* of medicine, the bedside teaching that medical students, residents and fellows should be learning. This individual will bring to the bedside their years of experience and thoughtful discussions of tough cases to make us all understand the basics of disease. You can inspire and create many more such Masters by your aura and presence on the floor.

In my career as a student and physician-in-training, the teachers who inspired me to become an Internist and a Nephrologist were all Master Clinicians and spent a lot of time with us - showing us physical examination skills, ways to think through tough cases and how to balance family and residency life. As a community of young physicians, we would like to extend an invitation to you to return and show to us your skills and enthusiasm. We understand the competing interests you have from administration, research and education. We can devise technological and novel ways of educating in the 21 ^st^ century to enable you to return. Advances in technology can aid in teaching clinical reasoning. As I walk through the hallways of the hospital, I realize what is absent… it is the “Master Clinician”. We miss you!

Sincerely,

*The Apprentice in search of a Guru*

