# Supplementary material for: A letter to the Master Clinician
Source: F1000Res. 2014 Feb 13;3:1. Originally published 2014 Jan 3. [Version 2] doi: 10.12688/f1000research.3-1.v2 (PMC3954161; doi:10.12688/f1000research.3-1.v2)
Supplement: Peer review history

# Peer review: Referee response for version 2

**Published:** 2014-02-17

**Authors:** Ritu Soni

**Affiliations:** 1 Division of General Internal Medicine, University of Pittsburgh, Pittsburgh, PA, USA

I have read this submission. I believe that I have an appropriate level of expertise to confirm that it is of an acceptable scientific standard.

# Peer review: Referee response for version 1

**Published:** 2014-02-10

**Authors:** Ritu Soni

**Affiliations:** 1 Division of General Internal Medicine, University of Pittsburgh, Pittsburgh, PA, USA

Very well written. I can identify with the ideas expressed in this article. In fact, it reminds me of my medical schooling in India, during which we focused on honing our clinical skills rather than relying on diagnostic imaging/procedures, due to limited resources. Our bedside rounds were more often than not painfully elaborate, with emphasis on physical examination. For instance, examination of the tongue used to be an hour-long lecture in itself. So were topics like variants of Babinski's reflex, types of pulses and their causes, dermatological findings in various diseases... and the list goes on. One would be fortunate to present his/her case beyond the physical examination part. 

 

But looking back, I miss that kind of learning. As much as I value my resourceful training in the U.S, I miss the mentorship of old-school clinicians, very appropriately addressed as Master Clinicians in the article. Hoping that these Master Clinicians don't go completely extinct in the future, and help us keep the art of medicine alive and vibrant.

My suggestions for revision:

- Consider deleting the lines- "While I support the advent of technology and the advancement of knowledge in medicine, medicine is also an art which cannot be taught by Google, Up-To-Date or Twitter. You have the experienced hands of a Master Clinician who carries the wisdom of diagnostic skills and carries knowledge." The same message is well conveyed in the paragraphs that follow.
   
- Consider consolidating the role of master clinicians into one paragraph and limiting the last paragraph to the 'invitation' alone, such that each paragraph conveys a distinct message.

An issue of concern, very creatively drafted.

I have read this submission. I believe that I have an appropriate level of expertise to confirm that it is of an acceptable scientific standard.

# Peer review: Referee response for version 1

**Published:** 2014-01-09

**Authors:** Laura Maursetter

**Affiliations:** 1 Department of Medicine, University of Wisconsin, Madison, WI, USA

A very nice idea to use this avenue to encourage the master clinician to spend more time where they will make a big impact. That is great. To me this type of publication needs to be short and sweet, to keep the interest in the topic and get the reader to continue to the end.  

I would suggest:

- Combine the first two paragraphs together.
- Keep the third paragraph as it is written - strong.
- The last 3 paragraphs seem to be repetitive - I would think through the ideas you are trying to convey. Spend time, inspire learner, teach skill-set to others - there are so many examples that I feel the message gets lost.

Overall, very nice. Thanks for the read.

I have read this submission. I believe that I have an appropriate level of expertise to confirm that it is of an acceptable scientific standard.
